# Assessment of extracranial carotid artery disease using digital twins – A pilot study

**DOI:** 10.1016/j.nicl.2023.103435

**Published:** 2023-05-13

**Authors:** Linus Dubs, Vasileios Charitatos, Stefano Buoso, Susanne Wegener, Sebastian Winklhofer, Hatem Alkadhi, Vartan Kurtcuoglu

**Affiliations:** a University of Zurich, Institute of Physiology, The Interface Group, Winterthurerstrasse 190, 8057 Zürich, Switzerland; b University Hospital Zurich, University of Zurich, Institute of Diagnostic and Interventional Radiology, Rämistrasse 100, 8091 Zürich, Switzerland; c ETH Zurich, Institute for Biomedical Engineering, Gloriastrasse 35, 8092 Zürich, Switzerland; d University Hospital Zurich, University of Zurich, Clinical Neuroscience Center, Department of Neurology, Frauenklinikstrasse 10, 8091 Zürich, Switzerland; e University Hospital Zurich, University of Zurich, Clinical Neuroscience Center, Department of Neuroradiology, Frauenklinikstrasse 10, 8091 Zürich, Switzerland

**Keywords:** Carotid artery stenosis, Computational fluid dynamics, Digital twins, Extracranial carotid artery disease, Fractional flow reserve

## Abstract

To improve risk stratification in extracranial internal carotid artery disease (CAD), patients who would benefit maximally from revascularization must be identified. In cardiology, the fractional flow reserve (FFR) has become a reference standard for evaluating the functional severity of coronary artery stenosis, and noninvasive surrogates thereof relying on computational fluid dynamics (CFD) have been developed. Here, we present a CFD-based workflow using digital twins of patients’ carotid bifurcations derived from computed tomography angiography for the noninvasive functional assessment of CAD. We reconstructed patient-specific digital twins of 37 carotid bifurcations. We implemented a CFD model using common carotid artery peak systolic velocity (PSV) acquired with Doppler ultrasound (DUS) as inlet boundary condition and a two-element Windkessel model as oulet boundary condition. The agreement between CFD and DUS on the PSV in the internal carotid artery (ICA) was then compared. The relative error for the agreement between DUS and CFD was 9% ± 20% and the intraclass correlation coefficient was 0.88. Furthermore, hyperemic simulations in a physiological range were feasible and unmasked markedly different pressure drops along two ICA stenoses with similar degree of narrowing under comparable ICA blood flow. Hereby, we lay the foundation for prospective studies on noninvasive CFD-based derivation of metrics similar to the FFR for the assessment of CAD.

## Highlights

• We present a workflow to assess the severity of carotid stenosis by digital twinning.
• Computed metrics showed good agreement with Doppler ultrasound measurements.
• Hyperemic simulations over carotid stenoses in a physiological range proved feasible.
• Digital twins may help in the development of biomarkers for stenosis assessment.

## 1 Introduction

Extracranial internal carotid artery disease (CAD) causes up to 15% of ischemic strokes (Flaherty et al., 2013, Kolominsky-Rabas et al., 2001). About a quarter of the adult population are affected by CAD, with approximately two in a hundred suffering from a degree of stenosis above 50% (De Weert et al., 2006, Song et al., 2020). This is accompanied by a 5-year-risk of ipsilateral stroke of up to 18% in asymptomatic patients (Howard et al., 2021). For asymptomatic patients with carotid artery stenosis >70% and for symptomatic patients with stenosis >50%, current guidelines recommend carotid revascularization along with best medical therapy (BMT) instead of BMT alone (AbuRahma et al., 2022).

While studies for symptomatic patients show reasonable evidence for the benefit of surgical revascularization (16% absolute risk reduction at 5 years, number needed to treat (NNT) = 6), the evidence for revascularization in asymptomatic patients is less convincing (Rothwell et al., 2003): In asymptomatic patients with >60% stenosis, the Asymptomatic Carotid Atherosclerosis Study showed an absolute risk reduction of 5.9% (NNT = 18) in the 5-year rate of ipsilateral stroke with BMT and carotid endarterectomy compared to BMT alone (Walker et al., 1995).

Therefore, it is evident that clinical decision making on the treatment of asymptomatic CAD must be improved, e.g., by identifying patients with increased risk of stroke and, therefore, higher expected benefit-to-risk ratio for revascularization. While correlation with outcome has been investigated for clinical features such as patient’s age, body mass index, comorbidities, contralateral disease, collateralization or plaque characteristics, the predictive value of hemodynamic characteristics in the stenosed vessel remains understudied (AbuRahma et al., 2022, Henderson et al., 2000, Keyhani et al., 2019, Murphy et al., 2020, Nicolaides et al., 2005). This is in stark contrast to coronary artery disease, where hemodynamic parameters such as the fractional flow reserve (FFR) have entered clinical routine and have revolutionized decision making in the management of myocardial ischemia (van de Hoef et al., 2013). The FFR describes the maximal blood flow over a stenotic artery as fraction of the maximal flow in the same artery in absence of stenosis (Pijls et al., 1993).

A direct transfer of coronary FFR to CAD is complicated by the fact that this metric is based on intravascular pressure measurements, which are associated with a periprocedural stroke risk of 1–4% for interventional cerebral angiography (Hankey et al., 1990). Moreover, an indispensable requirement of FFR is the induction of maximal flow. In fact, cerebral hyperemia is known to occur in situations of increased metabolic demand, acidosis, hypoxemia, hypercapnia or anemia, but standardized procedures for the induction of hyperemia for the purpose of FFR in CAD are lacking (Juttukonda and Donahue, 2019, Lin et al., 2010, Scheinberg and Jayne, 1952).

One potential noninvasive approach relies on a combination of medical imaging with computational fluid dynamics (CFD), a method for predicting pressures and velocities in fluids such as blood. This approach, also referred to as digital twinning, has been employed in hemodynamics research and has recently shown value for the noninvasive estimation of coronary FFR. The estimated metric is referred to as FFR_CT_ to reflect that it relies on computed tomography (CT) images (Coenen et al., 2015). While there are multiple studies that have focused on technical aspects of CFD models for evaluating CAD, there has been less published work on the translation to clinically validated tools (Lopes et al., 2020). Furthermore, previous studies have focused predominantly on workflows based on magnetic resonance imaging (MRI). Investigations based on CT angiography (CTA) with clinical focus beyond case studies are rare (Albadawi et al., 2021, Polanczyk et al., 2018). To our knowledge, there are no CTA-based CFD studies with large sample size and validation against measured data, even though CTA is a widely used, noninvasive imaging modality.

We hypothesize that metrics comparable in function to FFR_CT_ can be derived to assess the functional severity of extracranial carotid artery stenosis, thereby serving as a potential means for patient-specific risk stratification. A necessary first step towards that goal is the establishment and validation of a workflow for computing blood velocity and pressures in carotid bifurcations of patients. Herein, we present such a patient-specific workflow based on CTA and CFD. We performed a cross-validation of the computed velocities against Doppler ultrasound (DUS). We also investigated the effect of simulated hyperemic conditions on the relation between blood flow and pressure drop across stenoses, which is important for possible translation of the FFR metric to the evaluation of carotid artery disease.

## 2 Material and methods

### 2.1 Patient selection and ethics approval

We retrospectively screened the radiologic database of the University Hospital Zurich for stroke CT protocols conducted between June 8 2019 and November 3 2019 in clinical routine in patients with suspected stroke. This protocol includes a contrast-enhanced CTA of the head and neck vessels. Carotid bifurcations on both sides were considered if at least unilateral mild extracranial internal carotid artery (ICA) stenosis according to the NASCET criteria (Barnett et al., 1991, Naylor et al., 2018) was specified in the CTA report, regardless of the clinical presentation and outcome. Even in the absence of contralateral ICA stenosis, carotid bifurcations of both sides were used as controls for the CFD model. The study was approved by the ethics committee of the canton of Zurich (BASEC-Nr 2019–02428) and was governed according to the Declaration of Helsinki. Written general consent for research was given upon hospital admission. The required sample size was determined based on the intraclass correlation coefficient (ICC) as outcome measure (minimum acceptable ICC 0.75, expected ICC 0.9, α = 0.05, power 80%) (Bujang and Baharum, 2017). 60 carotid bifurcations of 32 patients (average age 73y, 81% male) with complete DUS reports were identified. 23 bifurcations were excluded because of insufficient image quality or artefacts (n = 8), dissection (n = 2), occlusion (n = 1), history of carotid endarterectomy (n = 4), and history of stenting (n = 1). Bifurcations with severe ICA stenosis were also excluded (n = 7), since the accuracy of DUS peak systolic velocity (PSV) measurement deteriorates substantially at high levels of stenosis (Mead et al., 2000). A total of 37 carotid bifurcations from 26 patients (average age 74y, 81% male) were thus further processed. Of the 37 carotid bifurcations included in the study, 7 ICAs were classified as ‘free of stenosis’, 24 as having ‘mild’, and 6 as having ‘moderate’ stenosis according to the DUS reports. Stenosis of the external carotid artery (ECA) was reported in 5 carotid bifurcations, and none for the common carotid artery (CCA). With the degree of ICA stenosis we refer to the severity determined by DUS reports according to adapted NASCET criteria (Naylor et al., 2018, Von Reutern et al., 2012), classifying stenoses as ‘free of stenosis’ for non-stenotic vessels, ‘mild’ for obstructions 10–49%, ‘moderate’ for obstructions 50–69%, and ‘severe’ for obstructions 70–99% (Grant et al., 2003, Naylor et al., 2018).

### 2.2 Image acquisition and reporting

Ultrasound was performed by different operators using various Philips (Amsterdam, the Netherlands) ultrasound devices with 9 or 18 MHz linear probes according to clinical routine protocols and by trained personnel. Extracranial carotid stenoses were described morphologically (B-mode), as well as according to the NASCET classification (Von Reutern et al., 2012). CT scans were performed on the standard emergency CT scanners of the department (X.cite or Somatom Definition Flash, Siemens Healthineers, Forchheim, Germany). The institutional standard protocol for patients with suspicion of stroke included a non-contrast scan of the head followed by an intra- and extracranial scan of the entire head and neck in an arterial contrast-enhanced phase.

### 2.3 Image processing

To generate three-dimensional anatomical models as the basis of digital twins, carotid bifurcations were segmented semi-automatically from the CTA images with a clinical post-processing software (Syngo.via, Siemens Healthineers, Munich, Germany). The carotid geometries included a CCA segment of approximately 5 cm. The ICA segments started at the branching of the CCA and ended at the entrance of the ICA into the carotid canal. The ECA segments extended for approximately 5 cm from the bifurcation and did not include side branches. Further flow extensions were added to the inlets and outlets to allow the flow to develop by the time it reached the arterial geometry. The validity of the extension approach has been confirmed by Hoi et al. under the condition that the length of the extension is at least three times the diameter of the CCA (Hoi et al., 2010).

### 2.4 Hemodynamic model

To enable flow calculations in the digital twins, vessel diameter-adaptive computational grids with tetrahedral elements were generated for each carotid geometry using the GMSH software (version 4.2.1) (Geuzaine and Remacle, 2009). Typical grid size ranged from 800,000 to 2,000,000 tetrahedral elements, with minimum and maximum edge lengths of 0.08 mm and 0.5 mm, respectively. The grids were considered stationary, meaning that transient deformation of the vascular wall was not considered. The assumption of rigid walls is accepted to have only a small impact on predicted velocities in the ICA (Lopes et al., 2019, Steinman, 2012). Blood was modeled as an incompressible Newtonian fluid with density ρ = 1060 kg/m^3^ and dynamic viscosity μ = 0.004 Pa^.^s, which is a reasonable assumption for hemodynamics in large vessels (Lopes et al., 2020, Morbiducci et al., 2010, Steinman, 2012). The unsteady three-dimensional incompressible Navier-Stokes equations were used to quantify blood flow as described previously (Buoso et al., 2022). At the vessel walls, a no-slip boundary condition was applied. At the inlet (CCA), we considered the blood flow direction to be normal to the inlet with a Poiseuille-type velocity profile $v_{\mathrm{in}}$:

(1) $$v_{\mathrm{in}}=v_{\mathrm{peak}}\left(1-\frac{x^{2}+y^{2}}{r_{\mathrm{CCA}}^{2}}\right),$$

where $v_{\mathrm{peak}}$ is the CCA PSV measured by DUS, x and y are the Cartesian coordinates of our domain and $r_{\mathrm{CCA}}$ is the CCA radius. This has been previously shown to be a reasonable choice for FFR_CT_ calculations (Buoso et al., 2022, Zhang et al., 2016). At the outlets (ICA and ECA), we applied a two-element Windkessel model relating blood pressure to blood flow rate (Westerhof et al., 2009):

(2) $$Q=\frac{P}{R}+C\frac{\mathrm{dP}}{\mathrm{dt}}$$

**Fig. 1:**
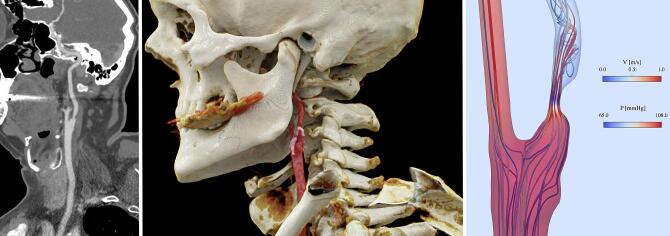
Overview of the workflow from computed tomographic angiography (CTA) to computational fluid dynamics (CFD). *Left*: The stenosis is identified in the CTA images and segmentation of the vessel lumen is performed. *Middle:* A 3D geometry is generated from the segmented images. *Right:* The geometry is meshed to a digital twin and CFD simulations are performed to calculate hemodynamic parameters such as blood pressure and velocity.

Here, Q is flow rate, R and C are the downstream microcirculatory resistance (peripheral resistance) and capacitance, respectively, and $\frac{\mathrm{dP}}{\mathrm{dt}}$ is the change in blood pressure over time. The Windkessel approach is commonly used in hemodynamic calculations to account for arterial compliance and the upstream vasculature’s resistance (Lopes et al., 2020). Here, the resistances $R_{\mathrm{ICA}}$ and $R_{\mathrm{ECA}}$ downstream of the ICA and ECA, respectively, are inversely proportional to the outlet vessel cross-sectional areas,

(3) $$\frac{R_{\mathrm{ICA}}}{R_{\mathrm{ECA}}}\propto {\left(\frac{r_{\mathrm{ECA}}}{r_{\mathrm{ICA}}}\right)}^{2},$$

where $r_{\mathrm{ECA}},r_{\mathrm{ICA}}$ are the ECA and ICA luminal radii, respectively (Murray, 1926). The ICA and ECA outflow pressures are calculated based on the Windkessel condition. We solved the Navier-Stokes equations using the open-source FEniCS finite element computing platform (version 2019.1.0) (Alnaes et al., 2015, Logg et al., 2012). Mesh independence was ensured by successive refinement of the computational grid until ICA PSV varied by <10% between consecutive grids. Postprocessing of the computed velocities and pressures was performed in a blinded fashion using the open-source visualization software ParaView (version 5.9.1). Fig. 1 depicts the workflow from CTA to the CFD model.

### 2.5 Validation of the workflow

We used ICA PSV to compare CFD with DUS, since PSV is readily available in patients’ files. The ICA PSV and the corresponding locations of the measurements were extracted from the DUS reports. We assumed that it is unlikely that operators placed the ultrasound acquisition window exactly at the center of the vessel, where the PSV is nominally expected, but with an offset of up to one ultrasound probe sampling volume (0.4 mm according to the manufacturer’s specifications). We corrected for this effect by adjusting the reported PSV based on the assumption of a parabolic velocity profile. In the following, the adjusted DUS values are reported. To examine the potential of our model to simulate the morphology-specific relationship between blood flow and pressure drop under hyperemic flow conditions, we selected two stenoses (Fig. 3), A and B, with similar degrees of stenosis (A: 47%, B: 51%). For the two stenoses, we computed blood flow and pressure distributions for three different blood flow conditions by varying CCA PSV in a physiologic range. Statistical analyses were performed using R studio (version 2022.02.1) based on standards of agreement analysis (Rothwell, 2000).

## 3 Results

### 3.1 Validation of the workflow

**Fig. 2:**
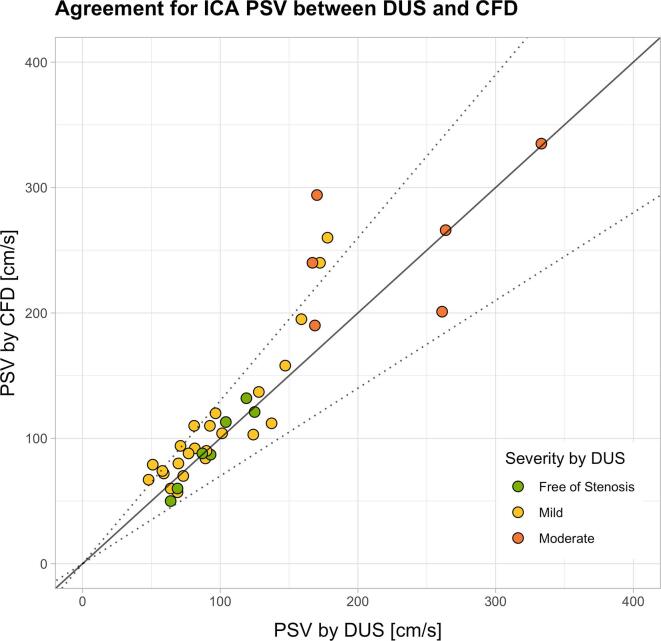

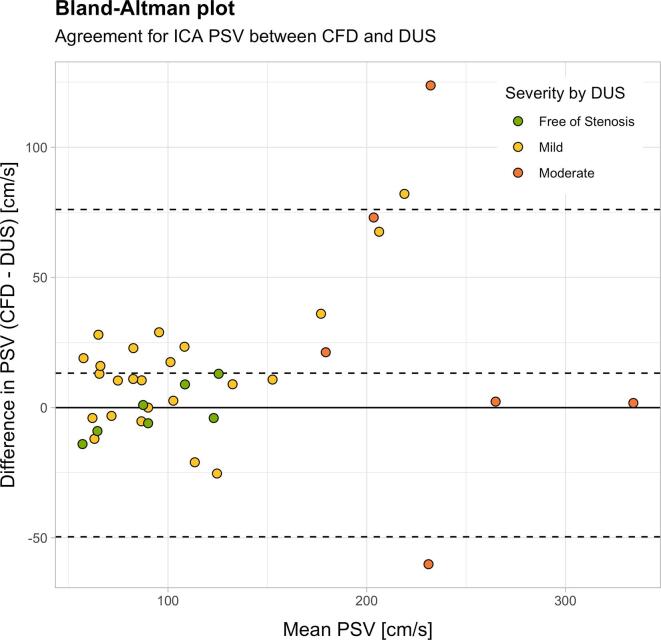
*Top:* Scatterplot depicting the agreement of peak systolic velocity (PSV) in the internal carotid artery (ICA) obtained by computational fluid dynamics (CFD) and Doppler ultrasound (DUS). The solid line corresponds to perfect agreement between modalities, whereas the dotted lines mark a ±30% margin of error. Colors indicate the classification of the degree of stenosis by DUS. *Bottom:* Bland-Altman plot to show the difference of PSV in the ICA obtained by CFD and DUS plotted against the mean PSV by both modalities. The solid line corresponds to zero, the dashed lines to the bias and the upper and lower limit of agreement.

To validate the established CFD workflow, we compared patient-specific ICA PSVs acquired by DUS and calculated by CFD. For assessing the agreement between the two modalities, we plotted the ICA PSV from DUS against values obtained by CFD (Fig. 2 top). The dotted lines define the ±30% error range reported in literature to be characteristic for DUS due to interobserver variability (Corriveau and Johnston, 2004). Values on the solid line (slope of 1) indicate perfect agreement between DUS and CFD. Measured ICA PSV values showed good agreement between modalities, with most of the measurements located within the expected error range. Linear regression analysis yielded the relation PSV_CFD_ = 8.3 cm/s + 1.042**^.^**PSV_DUS_ (R^2^ = 0.80, standard error for the slope: ±0.084), indicating a good agreement with slightly higher velocities predicted by CFD. A similar observation was obtained from the Bland-Altman plot in (Fig. 2 bottom), which indicated a bias of 13 cm/s, lower limit of agreement of −49 cm/s and upper limit of agreement of 76 cm/s. A good agreement was statistically verified by intraclass correlation, showing a correlation coefficient of 0.88 (ICC2, p < 0.001). The plot indicated worse agreement at higher velocities and correspondingly more advanced degree of stenosis. Overall, the average error as percentage of the mean of the measurements was 9% with a standard deviation of 20%. Whilst in the subgroup ‘free of stenosis’ the bias and limits of agreement, as well as the relative error and its standard deviation were −1 cm/s (−20 cm/s, 18 cm/s) and −4% (±12%), respectively, they were 27 cm/s (−98 cm/s, 152 cm/s) and 13% (±28%), respectively, in the ‘moderate’ subgroup. Table 1 gives an overview of these results. Hence, the agreement is in the expected range for all subgroups, but the systematic and random error increases absolutely and relatively with increasing degree of stenosis.

**Table 1:** Agreement between Doppler ultrasound (DUS) measurements and computational fluid dynamics (CFD) calculations of peak systolic velocity (PSV) in the internal carotid artery. Reported are mean velocity values ± standard deviation. n: sample size.

Severity by DUS	n	PSV_CFD_	PSV_DUS_	Δ PSV_CFD-DUS_	Δ PSV_CFD-DUS_ as % of mean PSV	Intraclass correlation coefficient
Free of stenosis	7	93 cm/s (±31 cm/s)	94 cm/s (±23 cm/s)	−1 cm/s (±10 cm/s)	−4% (±12%)	0.94
Mild	24	111 cm/s (±53 cm/s)	97 cm/s (±39 cm/s)	14 cm/s (±24 cm/s)	12% (±18%)	0.83
Moderate	6	254 cm/s (±56 cm/s)	227 cm/s (±69 cm/s)	27 cm/s (±64 cm/s)	13% (±28%)	0.48
Total	37	131 cm/s (±74 cm/s)	117 cm/s (±64 cm/s)	13 cm/s (±32 cm/s)	9% (±20%)	0.88

### 3.2 Potential use for patient-specific risk stratification

**Fig. 3:**
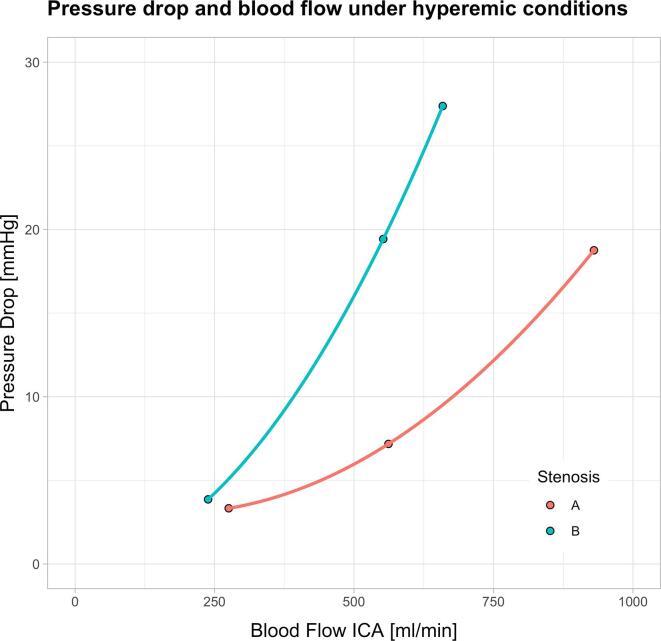
Visualization of computed pressure and velocity data on two different stenoses A (*top row*) and B (*bottom row*). *Left column:* Computed tomography angiography images. *Middle column:* Velocity and streamlines under steady state (*left*) and hyperemic conditions (*right*). *Right column:* Pressure drop along the stenoses under steady state (*left*) and hyperemic conditions (*right*).

**Fig. 4:**
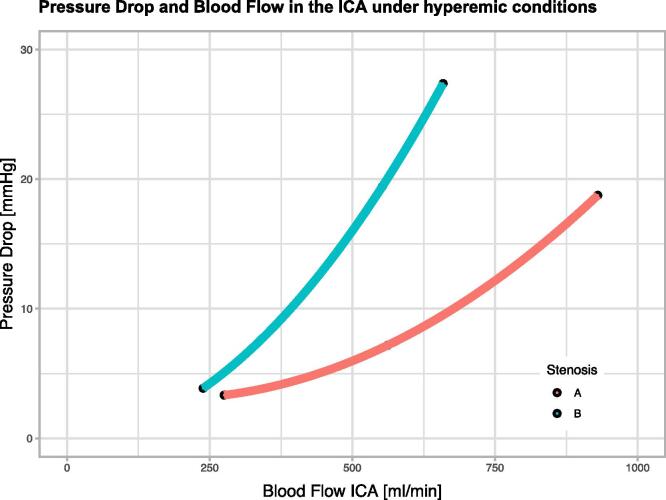
Pressure drop (ΔP) over the two stenoses shown in Fig. 3 as a function of increasing internal carotid artery (ICA) blood flow. Simulating hyperemia by progressively increasing blood inflow leads to a moderate increase in pressure drop across stenosis A, but a severe loss of pressure in stenosis B.

To illustrate that metrics comparable in function to FFR_CT_ can be derived from CFD for assessing the functional severity of carotid artery stenosis, we examined the potential of our model to capture the influence of morphology on the relation between blood flow and pressure drop under varying flow conditions. To this end, we selected two stenoses, A and B, with similar degrees of stenosis by diameter (A: 47%, B: 51%), and computed blood flow and pressure distributions for three different blood flow conditions by altering CCA PSV (Fig. 3). By increasing CCA PSV, hyperemic flow conditions are mimicked such as encountered in situations of increased metabolic demand. For stenosis A, ICA blood flow of 275 ml/min, 562 ml/min and 930 ml/min was associated with a pressure drop of 3 mmHg, 7 mmHg and 19 mmHg, respectively. For stenosis B, ICA blood flow of 238 ml/min, 552 ml/min, and 659 ml/min in the ICA was associated with a pressure drop of 4 mmHg, 19 mmHg and 27 mmHg, respectively. Fig. 4 shows how the pressure drop across the two stenoses develops with increasing flow. The pressure drop to flow relation can be approximated by a stenosis-specific second-degree polynomial function. Although the two stenoses have a similar degree of stenosis, their morphologic differences lead to an altered relationship of blood flow and pressure drop depending on the flow conditions. Whilst stenosis A shows a moderate increase in pressure drop over the stenosis under simulated hyperemia, stenosis B features a substantially larger pressure drop and, hence, becomes functionally more relevant in states of increased oxygen demand.

## 4 Discussion

We have established and validated a workflow for computing blood velocity and pressures in digital twins of carotid bifurcations of patients with carotid artery disease. This is a necessary first step towards establishing metrics comparable to CT-based fractional flow reserve for the noninvasive assessment of the functional severity of carotid artery stenosis. A total of 37 carotid bifurcations were analyzed, with Doppler ultrasound serving as reference for velocities calculated using computational fluid dynamics.

### 4.1 Agreement of velocity measured by Doppler ultrasound (DUS) with computational fluid dynamics (CFD)

Our results show a range of agreement from −49 cm/s to 76 cm/s for ICA PSV between DUS and CFD, with a bias of 13 cm/s. The average deviation as percentage of the mean of the measurements was 9%, with a standard deviation of 20%. This represents a reasonably good agreement with a mild bias. Liu et al. applied a CFD-workflow to 31 stenotic carotid bifurcations reconstructed from MRI and compared the results to DUS measurements (Liu et al., 2017). They report a slightly higher agreement with a bias of 9 cm/s and a range of agreement from −13 cm/s to 17 cm/s. However, their cases did not include PSV above 150 cm/s, where we observed increasing deviation between CFD and DUS. Considering the DUS interobserver variability for ICA PSV ranges from −68 cm/s to 85 cm/s or −25% to 43% as previously reported (Corriveau and Johnston, 2004, Mead et al., 2000), our range of agreement lies within clinically tolerated margins. Additionally, we observe that agreement between CFD and DUS is better for bifurcations characterized as ‘free of-stenosis’ and ones with ‘mild’ ICA stenosis compared to bifurcations showing ‘moderate’ degree of stenosis.

We attribute the overestimation of the velocity by 13 cm/s primarily to differences in the sampling between DUS and CFD. In CFD, the virtual measurement probe can be placed exactly at the center of the vessel, whereas this cannot be done in the same way with DUS – particularly not in a time-limited clinical setting. Furthermore, in cases where the ICA PSV is not located in the middle of the vessel (e.g., in curved segments), the highest velocity can be searched for and detected readily in CFD, whereas the same requires much more effort with DUS. At higher degrees of stenosis, we attribute the growing disagreement between CFD and DUS to the increase in the ultrasound probe sample volume relative to the vessel diameter and the larger effect of placing the probe even slightly off-center, since the radial velocity gradients increase with decreasing vessel diameter (Mead et al., 2000). In addition to differences in sampling, both modalities have their own inherent sources of error, which can lead to disagreements. For CFD, these include potentially inaccurate definition of the internal vessel geometry, which is dependent on the CTA image quality (reduced in the case of strong calcifications or dental implants). They also include simplifying assumptions for solving the hemodynamic equations, in particular considering vessel walls as rigid, neglecting non-Newtonian characteristics of blood, and choosing generic rather than patient-specific boundary conditions at the inlet (Xu et al., 2018). For DUS, uncertainty is introduced by inter- and intra-observer variability, differences in ultrasound machines, probes, and settings (sample volume, gain), poor imaging quality (in the presence of strong calcification, obese patients, tortuous vessels) and suboptimal probe angulation (Lui et al., 2005). Notably, in a clinical setting, the sonographic graduation of carotid stenosis not only relies on PSV but also on further parameters such as pre- and poststenotic flow or presence of collateralization.

### 4.2 Towards the functional assessment of extracranial carotid artery disease

Assessment of a stenosis by minimal diameter is fast and simple but does not necessarily reflect the clinical significance of the lesion accurately. This is illustrated by our analysis of the two exemplary cases A and B with similar stenosis by diameter, but markedly different functional severity during hyperemic conditions as revealed by CFD (Fig. 3, Fig. 4). A move away from purely geometric metrics towards hemodynamic ones for the evaluation of carotid artery stenoses is needed, just as FFR has become the reference standard for assessing the severity of coronary artery stenoses. The comparably high periprocedural stroke risk associated with interventional angiography has stood in the way of the adoption of FFR for CAD. In contrast, noninvasive surrogates based on CFD and CT, such as FFR_CT_, do not carry such risk and may prove of value for making clinical decisions on the treatment of asymptomatic CAD.

The assessment of pressure drop across a stenosis under hyperemic conditions is the basis for determining FFR. There have been previous studies aimed at computing such pressure drops in the carotid artery: Zhang et al. used CFD based on digital subtraction angiography in a single stenotic carotid artery and compared the calculated pressure gradient across the stenosis to the invasively measured spatial pressure difference (Zhang et al., 2018). Liu et al. derived pressure gradients from a CFD workflow based on MRI and showed that these correlated well with the degree of stenosis (Liu et al., 2016). However, neither study considered hyperemic conditions, which are fundamental for deriving FFR (Pijls and Tonino, 2011). Here, we have modeled hyperemia and assessed its impact on the predicted pressure drop (Fig. 3, Fig. 4).

### 4.3 Limitations

The single-center, retrospective format of this study accentuates some of the sources of error described above. Ultrasound examinations were performed by several different operators with a clinical rather than a research focus, thus introducing interobserver variability and imprecisions in clinically less relevant measurements. With 37 carotids, the sample size is limited. Further research on larger patient cohorts needs to be performed in the future, with a focus on clinical outcome for the purpose of establishing clinical biomarkers.

### 4.4 Conclusion

We have presented the validation of a workflow for computing blood velocity and pressures in carotid bifurcations of patients with CAD. Our results show that carotid artery blood velocities computed with CFD exhibit a level of agreement with DUS that justifies prospective studies for the derivation of metrics similar to FFR_CT_, but specific for carotid bifurcations.

## Resources of funding

We acknowledge financial support by a local grant from the Innovation Pool of the University Hospital Zurich and by the Swiss National Science Foundation through project 182683 and 310030_200703. The funding sources had no influence on the collection, analysis, and interpretation of data.

## CRediT authorship contribution statement

**Linus Dubs:** Conceptualization, Data curation, Formal analysis, Funding acquisition, Investigation, Methodology, Project administration, Validation, Visualization, Writing – original draft, Writing – review & editing. **Vasileios Charitatos:** Data curation, Formal analysis, Investigation, Methodology, Software, Validation, Visualization, Writing – original draft, Writing – review & editing. **Stefano Buoso:** Conceptualization, Formal analysis, Investigation, Methodology, Software, Writing – review & editing. **Susanne Wegener:** Funding acquisition, Project administration, Resources, Writing – review & editing. **Sebastian Winklhofer:** Project administration, Resources, Writing – review & editing. **Hatem Alkadhi:** Conceptualization, Funding acquisition, Project administration, Resources, Supervision, Writing – review & editing. **Vartan Kurtcuoglu:** Conceptualization, Funding acquisition, Project administration, Resources, Supervision, Writing – review & editing.

## Declaration of Competing Interest

The authors declare that they have no known competing financial interests or personal relationships that could have appeared to influence the work reported in this paper.

## Data Availability

Data will be made available on request.
